# Trust in healthcare: methodological and conceptual insights from mixed-method research in Lao People’s Democratic Republic

**DOI:** 10.1136/bmjgh-2023-014640

**Published:** 2024-05-15

**Authors:** Marco J Haenssgen, Elizabeth M Elliott, Sysavanh Phommachanh, Sylivanh Phomkong, Sengchanh Kounnavong, Shogo Kubota

**Affiliations:** 1 Department of Social Science and Development, Chiang Mai University, Chiang Mai, Thailand; 2 World Health Organization Regional Office for the Western Pacific, Manila, Philippines; 3 University of Health Sciences, Vientiane, Laos; 4 World Health Organization Representative Office, Vientiane, Lao People's Democratic Republic; 5 Lao Tropical and Public Health Institute, Vientiane, Lao People's Democratic Republic

**Keywords:** Health systems, Qualitative study, Other study design, Community-based survey

## Abstract

**Background:**

Global health foregrounds trust as a key requirement for the achievement of international health initiatives, but it remains an elusive concept that is often mobilised without consideration of its dimensions, drivers and downstream behavioural consequences. This paper aims to contribute to the conceptual development and measurement of ‘patient trust in primary healthcare’ from the lower middle-income country perspective of rural Lao PDR.

**Methods:**

A two-phase mixed-method research design was implemented between January 2021 and April 2023. Phase 1 involved exploratory qualitative research to understand the local expressions and dimensions of patient trust in primary healthcare, with 25 semistructured interviews and 17 focus group discussions (120 participants) in eight villages in Bokeo Province. Phase 2 involved explanatory research to assess patterns of trust systematically at scale in 14 villages across four provinces, wherein 26 cognitive interviews, 17 expert interviews and non-participant community observations informed a community census survey with 1838 participants. We analysed qualitative data through content-oriented thematic analysis and developed an 8-item trust scale on that basis. Quantitative data analysis used descriptive statistical and regression analysis.

**Results:**

We found that trust in primary healthcare is readily understood and intrinsically valuable in rural Lao PDR. Key dimensions included communication, respectful care, relationship, fairness, integrity, reputation, assurance of treatment and competence. The survey highlighted that reputation, competence, integrity and respectful care had the lowest trust scores. Health centre operations predicted the local expressions of trust. The behavioural consequences of trust were limited to a positive statistical association with antenatal care uptake among pregnant women but outweighed by alternative measures that also captured the availability of healthcare facilities.

**Conclusions:**

Overall, the development of our quantitative trust scale offers a process model for future researchers. We conclude that interpersonal, institutional and service-related trust require more explicit recognition in health system development and integration into health policy.

**WHAT IS ALREADY KNOWN ON THIS TOPIC**

- Global health research and policy foreground concerns about waning population trust in healthcare and science.
- Trust is a multidimensional concept, but its component dimensions are not well understood and research perspectives outside of high-income contexts are rare, which creates blind spots in literature and practice.

**WHAT THIS STUDY ADDS**

- We conducted original mixed-methods research to ground the understanding of trust in the local realities of a lower middle-income context.
- Trust in rural Lao PDR is readily understood, intrinsically valued and comprises eight distinct dimensions.
- The behavioural impacts of trust are mild and concentrated in antenatal healthcare access but can be leveraged relatively economically.

**HOW THIS STUDY MIGHT AFFECT RESEARCH, PRACTICE OR POLICY**

- We provide a methodology for developing and deploying locally grounded trust measurement at scale.
- Building interpersonal, institutional and service-related trust in healthcare can complement physical aspects of health system development but requires more attention through formal integration in health policies and initiatives.
- Trust-building interventions require substantial groundwork with target populations to identify key dimensions and issues in their trust towards health services.

## Background

### Introduction and objective

The notion of ‘trust’ has been argued to contribute positively to patients’ health service uptake, healthcare experiences, ‘better symptom-oriented subjective outcomes’ and even lower mortality in some cases.^1–3^ With renewed interest in the subject due to the rapid expansion of international health initiatives and the emergency responses to the COVID-19 pandemic, global health research and policy now foreground concerns about waning population trust in healthcare and science.^4–6^ However, trust remains an elusive notion that is often mobilised as a rhetorical device without consideration of its dimensions, drivers and downstream behavioural consequences.

The objective of this article is to contribute to the further development of the conceptual and methodological foundations of ‘trust in healthcare’. Drawing on the case of Lao PDR where exploratory sociomedical research has laid the foundation for a nation-wide initiative to enhance utilisation of rural primary healthcare services through community engagement, we address the question, ‘*What are the local expressions and patterns of trust in rural Lao PDR?’* In answering this question, we document a detailed mixed-methods approach to explore whether and how trust matters in local healthcare contexts of a lower middle-income country—a perspective that the literature has largely neglected. Our study, thus, identifies and illustrates eight distinct dimensions of trust in primary care services, and it offers a methodology to assess patterns, drivers and the behavioural implications of community trust in primary care services at scale.

### Conceptual and methodological background

‘Trust’ as either a noun or a verb is a broad concept with a wide range of meanings, making it complicated to define or measure.^6^ Pilgrim and Vassilev^7^ detail the multiple connotations of trust, which are subjectively experienced and implicitly related to power, risk and ethics. In healthcare, this includes both interpersonal trust in the healthcare provider, trust towards the product or service provided and systemic or institutional trust in the health system.^8 9^ Within the asymmetrical interaction with a healthcare provider, trust is inversely associated with the perception of risk by the patient and can, thus, be defined as ‘a set of expectations that the healthcare provider will do the best for the patient, and with good will, recognising the patient’s vulnerability’.^10^ As such, trust is subjected to not only external influencing factors such as health information or political action but also past health system operations and personal experiences therein.^8 9^

The most common method to measure trust in healthcare providers is a quantitative ‘trust scale’ that aggregates responses to a set of structured survey questions.^6 11^ A systematic review identified 45 different measures of trust in the health sector and 12 questions on average per scale.^12^ Among these measures, the most widely used is the ‘trust in Physician Scale’^13^—an 11-item self-reported instrument developed to assess an individual’s trust in their physician, for instance, by gauging agreement with such statements as ‘I trust my doctor (provider)’. However, broad assessments such as ‘I trust my doctor’ are likely to mask the reasons for good or poor trust (note that items in this scale as used, eg, by Anderson Dedrick,^13^ examine other dimensions of trust as well). Qualitative research can usefully complement quantitative work, for instance, by helping identify and develop contextually appropriate trust scales and their components. An example is the mixed-method approach of Greene and Ramos,^11^ who found that trust in US healthcare providers was highly correlated with the trust components of communication, caring and competence. A systematic literature review by Ozawa and Sripad^12^ documented in total eight such components, including honesty, communication, confidence, competence, fidelity, system trust, confidentiality and fairness.

Most studies in this field focus on high-income country contexts and especially the USA,^6^ the latter of which represented 37 out of the 45 studies, which Ozawa and Sripad^12^ reviewed (only three reviewed studies were not from high-income settings). Gopichandran *et al.* 14 argue that low and middle-income health systems commonly exhibit ‘deprivation of resources, lack of universal health access, low public expenditure on healthcare, high out of pocket expenditure on health and poorly regulated private practice’. As low and middle-income health systems thus differ systematically from high-income contexts (even where universal health coverage alleviates healthcare utilisation issues relating to affordability), they produce economically, politically, socially and also emotionally different health system encounters for local populations that afford further systematic research into the nuancing elements affecting trust in healthcare providers.

Among the rare examples outside of high-income settings is the qualitative study in rural south India by Gopichandran and Chetlapalli,^15^ which identified five key dimensions of trust that deviated from common trust scales in high-income settings, namely ‘perceived competence of the doctor, assurance of treatment (irrespective of time or ability to pay), willingness to accept drawbacks in the doctor, loyalty and respect’. Studies in Cambodia furthermore reported different levels of trust towards public and private health facilities, whereby private providers were deemed relatively more convenient, approachable, easy to contact and would accept delayed payments.^16 17^ Research from Mozambique found instead that the main influence on trust derived from healthcare providers’ communicative performances during their interactions with patients.^18^ These isolated yet rich examples demonstrate that more research in low- and middle-income contexts can help provide important nuance to the understanding of trust in healthcare.

In Lao PDR, qualitative research into healthcare-seeking experiences demonstrated that trust had strongly pronounced interpersonal components: people were found to select healthcare based on their relationship to, the reputation of, and recommendations by trusted people for the various local healthcare providers.^19 20^ Conversely, patients would be afraid of receiving poor care or paying extra fees if they did not have any previous connection with the provider.^21^ Other qualitative and quantitative studies have further shown that trust in providers was also affected by the providers’ communication and counselling skills as well as their attitudes.^22–24^ Providing services that people want and need, and regular interaction with the local community, was also argued to encourage healthcare utilisation and to build trust from both a service and institutional perspective.^25^

## Methods

### Patient and public involvement

The research took place across rural Lao PDR and in conjunction with a nationwide initiative to strengthen primary healthcare services that the Lao Ministry of Health (MoH) and Ministry of Home Affairs (MoHA) implemented with technical support from the WHO.^26^ This initiative was borne out of healthcare challenges experienced during the COVID-19 pandemic and involved support for localised healthcare governance and a multisectoral approach to empower communities to jointly improve health of the people in Lao PDR.^26^ Relationship building and healthcare ownership through trust-building engagement activities on the village level played a central role in this process; the initiative became accordingly referred to as Community Network Engagement for Essential Healthcare and COVID-19 Responses Through Trust (CONNECT)—and motivated the present research. The roll-out of CONNECT commenced in December 2021 (and was still ongoing at the time of manuscript submission), targeting high priority ‘focus’ districts and villages nation-wide. The foundational research reported in this manuscript, therefore, constituted a critical form of public involvement in the design and implementation of the CONNECT initiative. As we further indicate in the Results sections, we elicited and received positive participant feedback about the data collection process and research instrument.

### Research sites

The study setting of rural Lao PDR represents a lower middle income country context with rapid development progress yet persistent healthcare challenges and inequalities.^27^ Official poverty headcount rates (at US$2.15/day in purchasing power parity) declined from 25.4% in 2002 to 7.1% in 2018, and between 2000 and 2020, life expectancy increased from 58 to 68 years and rural electrification from 27.4% to 100%.^27^ Despite this progress, public health expenditure has remained at a comparatively low 1.2% of gross domestic product, and its 42.9% share of total health expenditure hardly exceeded the 41.8% accounted for by out-of-pocket expenses in 2020.^27^ Lao PDR is also still dependent on health aid, which accounted for 15.4% of total health expenditure in 2020.^27^ In addition, 63.1% of the country’s 7.4 m population live scattered across the vast rural areas (Lao PDR is the most sparsely populated country in Southeast Asia and the seventh in all Asia), which make infrastructure and healthcare provision challenging.^28^ For example, safely managed drinking water access remains at a low 12.4% in 2020 while latest available data from 2017 indicate that only 67.0% of births in rural areas were registered.^27^ Rural healthcare provision, therefore, remains challenging, even if recent advances in National Health Insurance provision as de facto universal health coverage scheme have lowered the costs of accessing health services to a nominal copayment of LAK 5000 (US$ 0.30).

**Figure 1:**
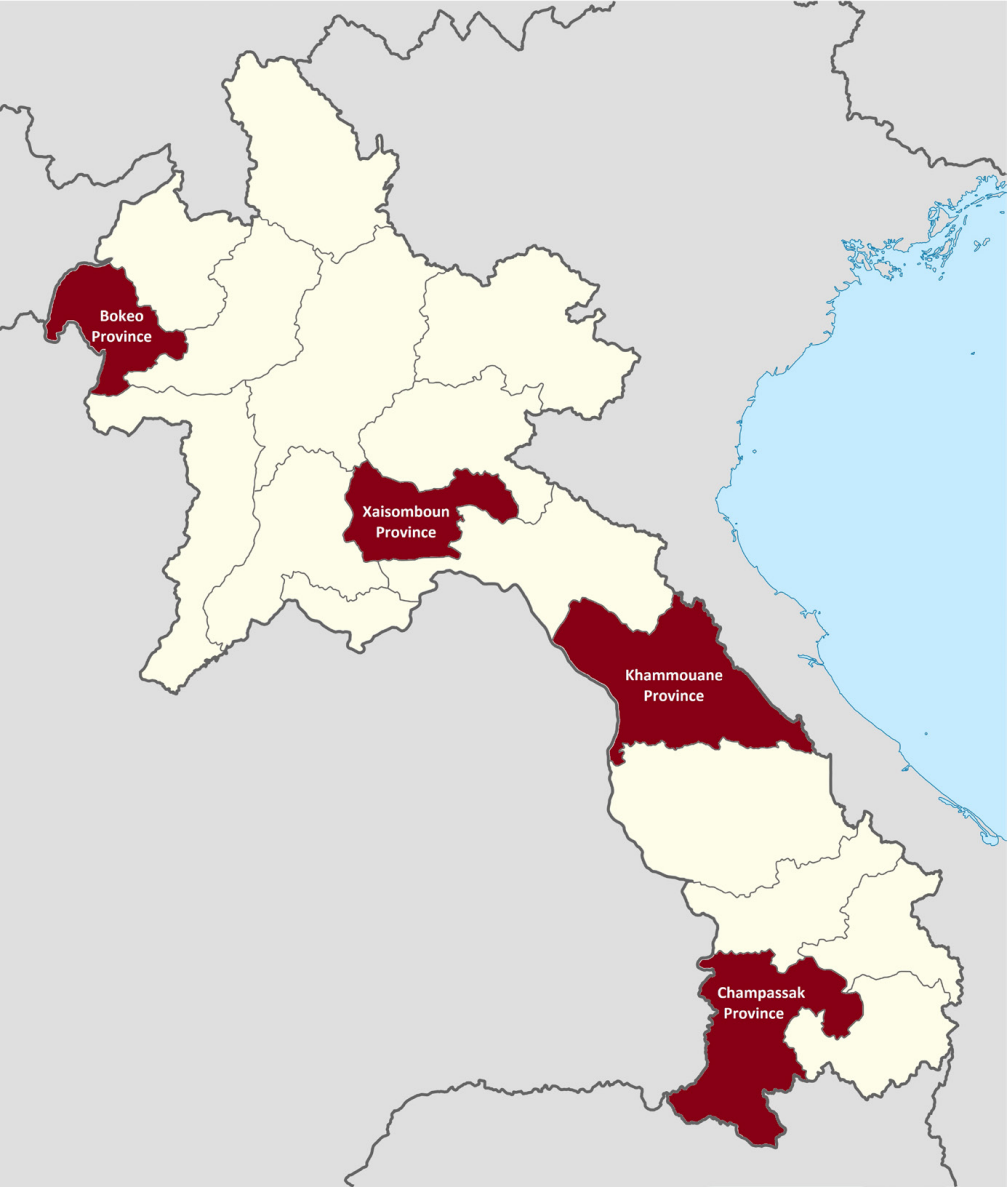
Map of Lao PDR and study sites. Source: Adapted from *Wikimedia Commons* (https://commons.wikimedia.org; file: Laos provinces.svg. user: Infernoapple). Study provinces highlighted in red; Bokeo province was also site for the preliminary qualitative research.

The specific study sites were the provinces of Bokeo, Xaisomboun, Khammouane and Champassak (figure 1). Latest poverty measurement data by Coulombe *et al.* 29 from the year 2015 indicated that Bokeo had a poverty headcount ratio of 25.5% and an adult literacy rate of 67.0%, with corresponding values in Xaisomboun of 27.8% and 74.4%, in Khammouane of 27.1% and 83.5% and in Champassak of 22.8% and 91.2%. While Bokeo Province was also the site for the preliminary research, all provinces together represented a selection of geographically, economically and ethnically diverse settings.

### Research design

**Figure 2:**
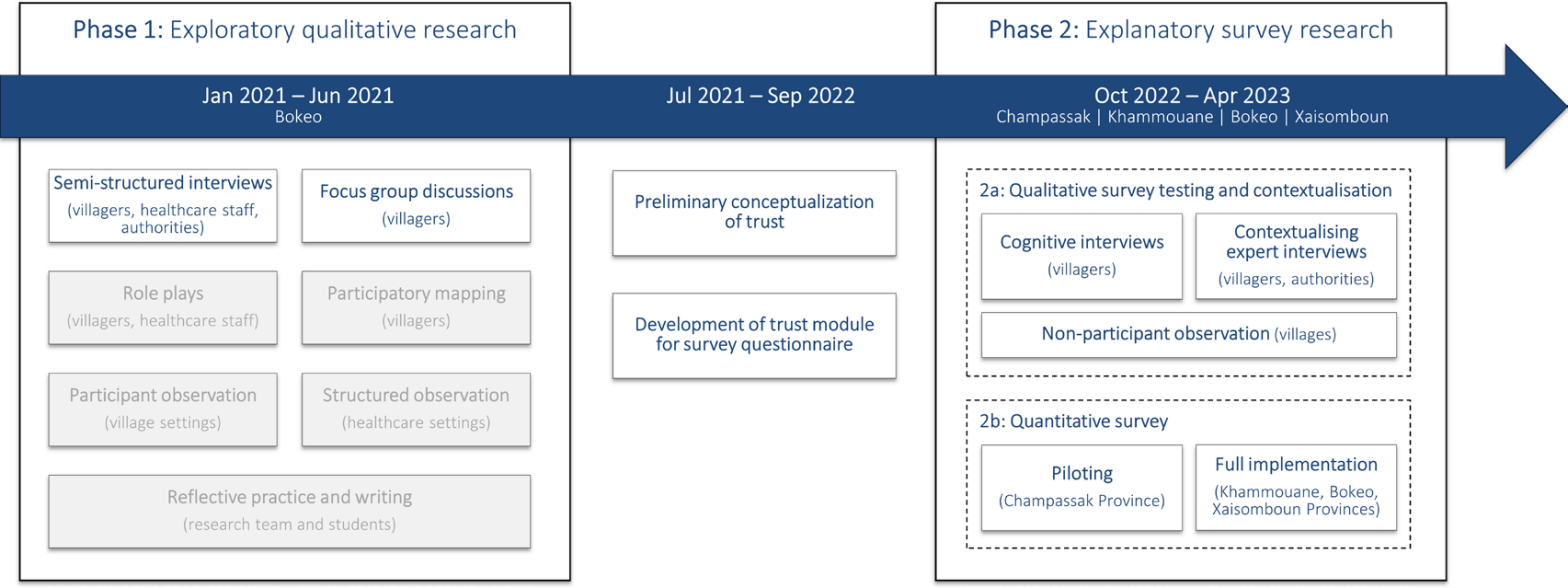
Mixed-method research design including timeline and data collection tools. Source: Authors. Grey-shaded elements were part of the larger preliminary research for the CONNECT Initiative but did not inform the research objective on trust. CONNECT, Community Network Engagement for Essential Healthcare and COVID-19 Responses Through Trust.

Our research team implemented a sequential mixed-method research design to study the local expressions and patterns of trust in rural Lao PDR (figure 2). Phase 1 aligned with the CONNECT Initiative and involved exploratory qualitative research that aimed at generating evidence to improve community engagement, to increase uptake of essential maternal and child healthcare services and to develop community-led COVID-19 responses. The second phase translated the qualitative findings into a survey questionnaire with a dedicated module on trust and involved a parallel qualitative–quantitative design: phase 2a tested the survey instrument through cognitive and contextualising expert interviews, and phase 2b piloted and implemented the survey questionnaire.

### Data collection

Data collection during phase 1 took place in February 2021 in Bokeo Province. Among the broader range of open-ended qualitative data collection methods during this phase,^30^ those that enabled a specific insight into the issue of trust from community members’ perspectives were 30–60 min semistructured interviews and 90 min focus group discussions with villagers. The interviews and focus group discussions were convened by trained master’s students and staff from the Lao Tropical and Public Health Institute (Lao TPHI) with support from the University of Health Sciences (UHS), Mother and Child Health Centre and WHO in Lao PDR. Each interview and discussion session was audio recorded with prior informed consent. In the focus groups, one investigator led the session through a series of open-ended questions and prompts while other investigators noted key points and observations, which were subsequently synthesised through daily discussions among the team in the field to draw out common themes.

The open-ended approach (summarised here to avoid redundancies and to retain brevity of the overall manuscript) indicated that key reasons for poor trust in health providers included: being charged extra fees or being told to buy medicines privately; nobody available at health facility or no medicines/equipment; own or peers’ bad experiences; healthcare staff speaking impolitely, not paying attention, or having difficulties in communicating needs; not having any previous contact/connection with health staff; difficulties with understanding or using health insurance, lack of willingness by health staff to be flexible about documentation; health staff from a different gender/ethnic group, fear of discrimination or embarrassment. These initial insights formed the basis for a preliminary survey questionnaire module on community trust in primary healthcare services. The process was supported by the grounded inputs from the Lao research teams (who specialised in community development) and the expert review by Lao health systems and maternal and child health experts as well as medical anthropologists within our team.

Phase 2 took place from October 2022 to April 2023 and involved the piloting and implementation of the tablet-based and interviewer-administered 40 min survey questionnaire and its 10 min module on community trust in their local health centres. The pilot took place in Champassak Province and was followed by a further round of expert review, which entailed refinement of the question focus (eg, considering that payments and gifts may be voluntary to express gratitude), the range of indicators (ie, removing systems trust) and the answer categories (eg, capturing indifference in yes/no answer options). Both the pilot and implementation stage were accompanied by 20–30 min cognitive interviews to help develop the questionnaire and interpret its data (we used an open-ended variant of cognitive interviews that resembled more natural semistructured conversations on the individual survey items and thereby accommodated the interaction dynamics more respectfully in the local context than the structured ‘think aloud’ process of the original cognitive interview conceived in Western contexts), and 30–45 min expert interviews with community members, village authorities and health centre staff plus non-participant observations of community life to provider broader community context for survey data interpretation.^31^ The data were collected by a seven-member Lao survey team experienced in community development, who gathered responses in Lao or in the preferred ethnic languages of the respondents (for which we recruited local translators). The data collection instruments are available in online supplemental material.

10.1136/bmjgh-2023-014640.supp1 Supplementary data

### Sampling

Fieldwork for phase 1 was conducted in two districts of Bokeo province. In each district, the research team purposively selected two health centres based on variation in healthcare service uptake (informed by administrative data), and two villages in their catchment areas with varying sociodemographic characteristics (ethnicity, livelihoods, income). Across these four health centres and eight villages, 25 people participated in the semistructured interviews, including 11 pregnant women and 14 village health committee members (all aged between 18 and 55 years). A further 120 people joined 17 focus group discussions with five to eight homogenous participants each, covering six groups with pregnant women, six groups with husbands and five groups with senior villagers aged above 60 years. The participants had Hmong, Khmu, Lue, Lamed and lowland Lao ethnicity.^30^

Data collection in phase 2 took place in three-stage survey design with non-probabilistic sampling. In the first stage, this involved four purposively selected provinces to represent diverse social, economic and healthcare settings across Lao PDR and following loosely the gradual roll-out of the CONNECT Initiative. Following the CONNECT objectives of reaching marginalised communities who experienced challenges in maternal and child healthcare uptake, the second stage involved the purposive selection of 14 CONNECT target villages as study sites, which were selected with variable levels of primary healthcare service uptake (prioritising low-uptake settings), remoteness, village size and ethnic diversity. Within the study communities, the third stage (representing study phase 2b) involved a complete census of all villagers (inclusion criteria: aged 16 and above, ordinary resident of the selected community). In the absence of comprehensive village registers prior to the data collection, we established sampling frames from publicly available satellite imagery to identify, list and approach all residential structures in a community, and update them dynamically during the data collection process where satellite images did not yet include newly built houses.^32^ The resulting survey samples, thus, reflected a snapshot of the daytime resident population in the study communities with between 43 and 318 responses per village (131 on average) and 1838 responses in total (table 1; refusal rates were below 5% on average). Complementary qualitative research took place alongside the survey (phase 2a) and involved a purposive selection of cognitive interview participants and expert informants based on their healthcare experiences, gender and their reported experiences of trust during the quantitative survey. The expert interviews involved village authorities and healthcare staff. The ensuing 26 cognitive interviews and 17 expert interviews included 31 villagers and village authorities, of whom 45.2% were women with ethnic groups spanning Hmong, Khmu, Lamed, Phu Thai, Jalee and lowland Lao. Online supplemental material table A1 provides further summary statistics on the demographic attributes of the sample.

**Table 1:** Summary of qualitative and quantitative samples

	Province				Total
	Bokeo	Champassak	Khammouane	Xaisomboun	
Phase 1					
Semistructured interviews	25				25
Focus group participants	120				120
Phase 2a					
Cognitive interviews	4	5	7	10	26
Expert interviews	5	3	5	4	17
Phase 2b					
Survey interviews	440	658	222	518	1838

Source: Authors.

### Analysis

Phase I qualitative data were transcribed into Lao and analysed by the Lao-speaking members of our research team (led by Lao TPHI, UHS, and WHO). For phase II, we transcribed and digitised all qualitative material and maintained bilingual Lao and English versions, which our broader study team analysed bilingually.^31^ Analysis was carried out using spreadsheets and MAXQDA 2020^33^ and following content-oriented thematic analysis. In phase I, this analysis process was inductive and involved identifying and coding any trust-related content of the data and subsequently categorising it into patterns or themes related to the various components of trust, so as to arrive at a comprehensive understanding of the varied dimensions of trust in primary healthcare services. The qualitative thematic analysis process in phase II was instead deductive as it focused on the initially established themes in order to identify their expressions and relevance as well as to triangulate the phase I findings. In this process of data triangulation across phases, the diversity of respondents and settings helped to actively source negative cases to challenge—and more clearly delineate—the categories within the evolving structure of the multidimensional trust concept. With view towards conciseness, the presentation of the qualitative findings in this manuscript will thereby foreground the responses to the cognitive interviews as they provide direct reactions to the preliminary trust dimensions developed during Phase 1 (on the basis of broader, open-ended qualitative data).

The subsequent quantitative analysis aimed at presenting the (a) resulting patterns, (b) socioeconomic correlates and (c) behavioural consequences of trust in rural Lao PDR. To describe the levels and patterns of trust and its component dimensions, we used non-inferential descriptive statistical analysis given that the community census data already represented the daytime adult population in the 14 case study communities.^34^ The descriptive analysis in step (a) thereby served as an additional layer of method triangulation to establish the prevalence and emerging patterns of trust among our diverse study sites in Lao PDR. Steps (b) and (c) used multivariate regression analysis,^34^ namely: linear regression models to understand the predictors of the trust index (using common socio-economic indicators such as gender, education, wealth, ethnicity and village location), and logistic regression models to understand the contribution of trust to health centre access during acute illnesses (focusing on illness episodes reported by adults and for children under their supervision) and during pregnancies (reported by currently and recently pregnant women). The regression models in step (c) both controlled for standard socioeconomic indicators; healthcare access during acute illnesses further controlled for illness severity and whether the patient was an adult or child. Although the pilot phase entailed slight modifications to the original survey instrument, the trust indices in the pilot and full-implementation provinces were highly correlated and we, therefore, present provincial-level disaggregated statistics from all four provinces in this paper.

### Ethical considerations

Prior informed consent was obtained from all study participants. The data collection in both phases was integrated into government structures: public health authorities facilitated village access and data collection; operational insights from each survey mission were fed back to village and health authorities; and survey findings were shared with provincial and central government authorities (including MoH and MoHA).

## Results

### Qualitative exploration

#### Relevance of trust

Before exploring what trust means concretely, it is helpful to establish its relevance in the rural Lao context. The qualitative findings suggested that it did: participants were generally comfortable talking about trust, using the typical words (or ethnic language equivalents) for the literal Lao translations of ‘trust’ (ຄວາມໄວ້ເນື້ອເຊື່ອໃຈ or *khuam vai nuea suea jai*) and ‘confidence’ (ຄວາມໝັ້ນໃຈ or *khuam mun jai*). As a widely understood concept, participants underlined that ‘*trust is important’* (man, 42, lowland Lao, Khammouane Province, cognitive interview) and were easily able to explicate their trust in primary healthcare services, explaining, for instance, that they had ‘*about 70%’* confidence in their local health centres (man, 48, Hmong, Xaisomboun Province; cognitive interview). Even if the issue of trust was not being probed explicitly, it arose naturally during conversations and observations.

The subject of trust also appeared to have often real implications for the treatment-seeking behaviour of patients and caregivers as villagers would in low-trust situations ‘*skip the health centre’* (woman, 26, lowland Lao, Champassak Province, cognitive interview) and ‘*better go directly to the district hospital’* (man, 37, Phu Thai, Khammouane Province, cognitive interview). Trust was, therefore, a pervasive and relevant concept in local treatment-seeking practices.

#### Defining trust

Participants were able to articulate personal definitions of trust, for instance, relating to health centre staff doing *a very good job’* so that *the patients will get better very fast* (man, 42 and 37, lowland Lao, Khammouane Province) and in recognition of the efforts and intrinsic motivation of healthcare workers to use of *their ability to help people* (man, 52, Khmu, Bokeo Province; similar statements were also encountered among Khmu and Hmong respondents in Xaisomboun Province). Ethnic minority groups further stressed in their definitions of trust the direct interactions with healthcare staff, such as in the case of a 24-year-old woman Lamed villager: *the doctors do not pay close attention to us. I think just because I am not the head of the village or someone that important, they do not treat us well*.

However, trust was not explicitly recognised among every villager. Especially female respondents would reiterate that they cannot explain the idea of ‘trust’ and that *It’s just that I don’t understand it’* (woman, 21, lowland Lao, Khammouane Province; woman, 27, Khmu, Bokeo Province; woman, 25, Hmong, Xaisomboun Province). Trust does also not automatically supersede other pragmatic considerations of healthcare utilisation such as mere availability of facilities and medical supplies. A 64-year-old lowland Lao man in Champassak Province would share in this context that he *absolutely* trusts the local health centre, but *if something happened here, we are likely to go to the 103 Hospital* (a local military hospital)*, which is closer to us than the health centre*.

Overall, this wide range of definitions suggests that externally assigned definitions of trust run the risk of misrepresenting local populations’ priorities. A systematic consideration of the components of this concept is, therefore, beneficial to create locally relevant forms of measurement.

#### Trust components in rural Lao PDR

**Figure 3:**
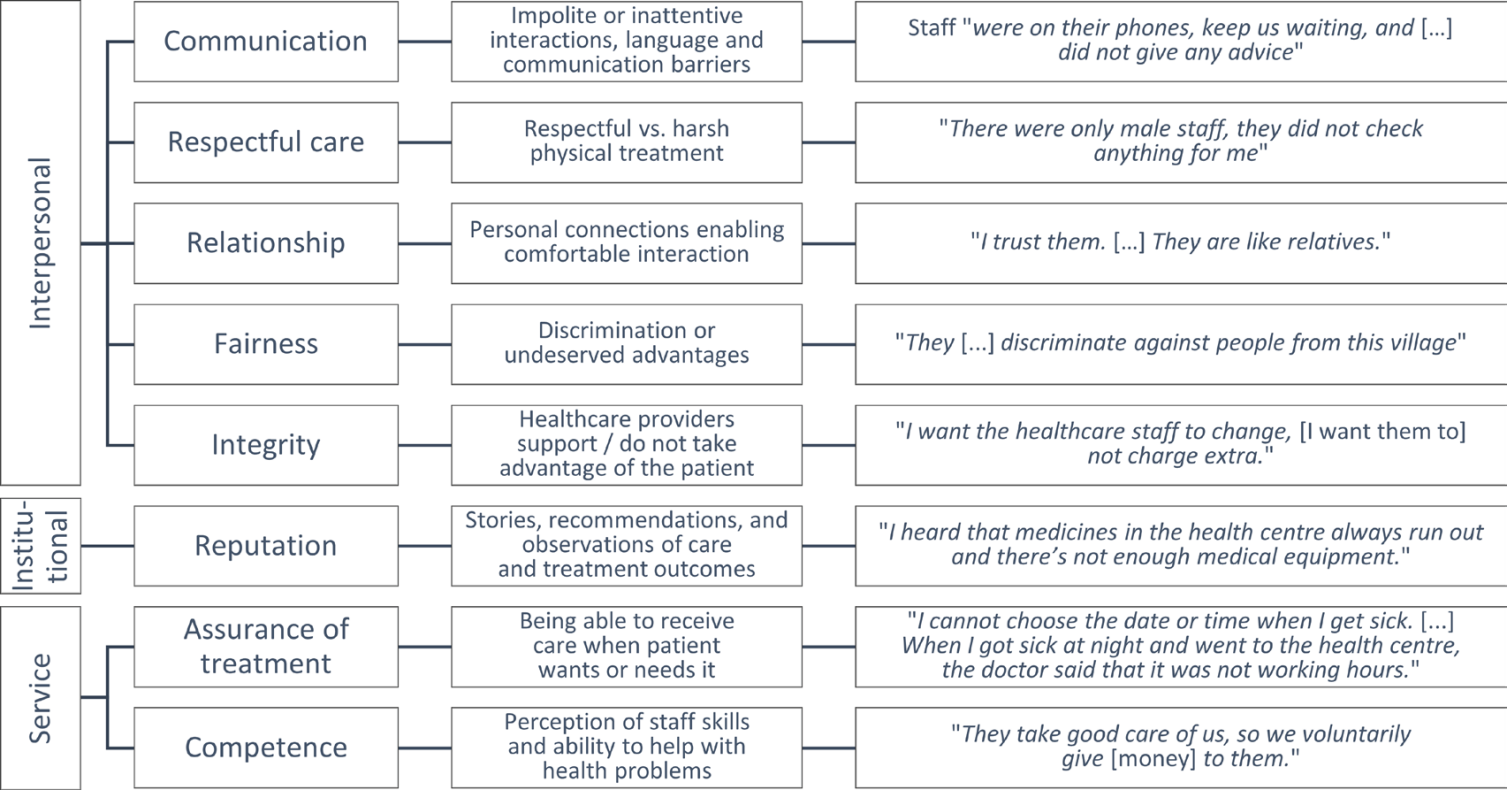
Coding framework representing categories of trust as core themes, with explanation and example quotes. Source: Authors, derived from qualitative research fieldwork.

The qualitative analysis yielded eight themes representing subcategories of trust in primary healthcare services in rural Lao PDR across the three main types of trust—interpersonal, institutional and service-related trust (represented through the coding framework in figure 3), which formed the basis for the subsequent development of quantitative survey indicators to measure trust at scale.

The first theme was *communication* as a form of interpersonal trust, with impolite, inattentive or difficult interactions indicating diminished trust in the provider. Villagers were generally ready to articulate their opinions about communication experiences. While experiences sometimes involved nuanced statements or even explicit praise (eg, *They were polite and explain the symptoms, treatment, and recommendation;’* man, 47, Jalee, Khammouane Province, cognitive interview), it was mainly the negative communication experiences that participants related directly to their trust in healthcare staff. One of several examples involved a respondent in Champassak province, who recalled situations where staff *were on their phones, keep us waiting, and* […] *did not give any advice* (man, 29, lowland Lao, Champassak Province, cognitive interview). Also language barriers would provoke experiences of abrasive interactions and poor treatment—as especially female members of ethnic communities reported repeatedly.

A second and related component of trust was the concrete treatment experience, represented through *respectful care* (ie, interpersonal trust, owing to the emphasis on ‘respect’). Common experiences of disrespect arose during maternal and child care at the health centres. In one such case, a woman bringing her feverish child to the health centre found only male staff who ‘*did not really care, just do everything very quickly’* (woman, 26, lowland Lao, Champassak Province, cognitive interview). Likewise, a mother delivering her baby at the health centre recalled how staff ‘*did not do anything. They just cut the umbilical cord, but did not clean the baby, and then they gave the baby to my mother-in-law’*, during which process she was left exposed as the staff ‘*did not cover up anything for me’* (woman, 24, Lamed, Bokeo Province, cognitive interview). Treatment experiences, therefore, often related negatively to trust; that is, they were disrespectful.

A third component of trust was the nature of the personal *relationships* between community members and health centres (interpersonal trust). Existing connections, a shared community identity, or outreach activities would create familiarity and enable comfortable interactions (thus closely relating to the two aforementioned dimensions). Such relationships were typically expressed in positive terms like, *Every time I go to the health centre, I can talk comfortably to the staff* (man, 37, lowland Lao, Khammouane Province, cognitive interview; note that this is a typical Lao expression to describe a familiar relationship) ‘*They are like relatives’* (man, 53, lowland Lao, Champassak Province, cognitive interview). Positive relationships were also forged through health centre outreach activities, for example, to administer medicine and food supplements for children or to donate clothes (man, 47, Jalee, Khammouane Province, cognitive interview).

The fourth theme was *fairness* (interpersonal trust), which often followed as practical consequence from the relationships between health centres and communities. Expert interviews with village authorities and health centre staff would normally stress that all people were treated equally (eg, health centre director, Bokeo Province; village chief, Bokeo Province), but villagers’ views were more diverse (although some villagers agreed). Villagers in Champassak Province described for example how kinship ties with healthcare staff enabled preferential treatment, Bokeo villagers reported that health centres provide systematically better care for those villages where their staff live, and experiences of wealth-based and ethnic discrimination arose across the provinces. During our community visits in Bokeo, a villager shared, for instance, how she saw health centre staff neglecting poorly dressed patients, while another villager from a Lamed community experienced that the health centre staff *discriminate against people from this village* because they were *not like Thai Lue* (like the health centre staff) *or lowland Lao* (woman, 24, Lamed, Bokeo Province, cognitive interview).

The fifth component of trust was *integrity* (interpersonal trust), which related to a supportive healthcare environment in which providers behave transparently and without taking advantage of patients. Aside from perceptions of honesty and support through the care experience at the health centre (eg, with documentation), the key manifestation of integrity was whether healthcare workers explicitly demanded or implicitly valued payments beyond the official costs of healthcare provision—directly in cash or by requestion patients to buy out-of-stock medicines privately at the houses of health centre staff. This issue was most salient in the interviews in Khammouane and Bokeo Provinces, and preliminary research participants in Bokeo Province would repeatedly stress this problem:

*When I go to the health centre, the staff asks me the question, ‘Did you bring a lot of money or not?’ If I have money, they would treat me, but if I don’t have money, they would tell me to go to another hospital* (pregnant woman, Bokeo Province, preliminary focus group discussion).

Taking a broader institutional perspective, the sixth component of trust was the *reputation of the healthcare provider*. Indeed only few villagers ‘*never heard any* (stories)’ (man, 53, lowland Lao, Champassak Province); rumours and narratives were pervasive in all communities. A typical kind of story would relate to treatment failure: A Champassak villager shared that her friends delivered a baby at her local health centre, but she lost too much blood and got transferred to the state hospital and eventually did not survive. Such and other outcomes like the death of a child would lead villagers to no longer trust the local health centre (woman, 26, lowland Lao, Champassak Province, cognitive interview). Elsewhere rumours spread that healthcare staff were illegally charging LAK 50 000 (approximately US$2.50) per COVID vaccine injection after claiming that they were out of stock. Rumours and stories thus typically undermined health providers’ reputation, and only rarely involved positive reports and recommendations.

The remaining two components both reflected service-related trust, the first of which was the *assurance of treatment*. This component reflected patients’ ability to receive care when they want or need it. Staffing, availability of medicines and also acceptance or refusal of patient requests would shape how community members trusted their health centres. A pregnant woman during a preliminary focus group discussion in Bokeo province illustrated this problem vividly through the case of a fellow villager:

*A pregnant woman visited the health centre and did not see any staff there. Then she looked for them at the back of the health office, but the staff got upset and shouted at the woman, saying that, ‘At your age, you already had experience of pregnancy. So why do you come here?’ That is why we do not like going to the health centre except when we have a severe illness, otherwise we do not go. Even if we go there, we get nothing from them*. (Pregnant woman, Bokeo Province, preliminary focus group discussion)

Such examples of trust-impacting assurance of treatment were common, and also included cases such as absent health centre staff who were instead found drinking beer (Champassak Province) or long queues and delays in getting access during emergencies (Bokeo Province).

The final service-related dimension of trust pertained to the perceived *competence* of the health centre staff. Healthcare staff skills featured prominently in community members’ narratives, whereas questions surrounding the assurance of treatment were often answered in negative terms, perceptions about staff competence where typically more balanced, with positive impressions often underlined by patients’ willingness to voluntarily offer additional compensation to staff: *They never asked* (for money). *But we offer them* (money) *as a gift. They take good care of us, so we voluntarily give it to them*. […] *If we don’t give them money, they would still take good care of us* (man, 44, Hmong, Xaisomboun Province, cognitive interview).

In addition to these eight dimensions, the literature and the preliminary research phase would also highlight ‘systems trust’ as an important institutional component of trust that goes beyond the direct relationship between community members and health centres. Systems trust offers a useful perspective that could complement the existing institutional theme of ‘reputation’ in our qualitative research. However, systems trust as a broader belief in institutions, processes and policies of the health system (eg, the government’s ability to establish a functional referral system, see Straten *et al* 35) often remained an abstract idea for villagers or would otherwise materialise in expressions that were orthogonally related to trust in primary health services. Villagers would, for instance, describe that needing to access a health centre first to get a referral to a district hospital would be *an inconvenience* (woman, 26, lowland Lao, Champassak Province, cognitive interview) as they would *go there just to waste time and money for fuel* (man, 37, Phu Thai, Khammouane Province, cognitive interview)—rather than expressing their trust in the dependence and reliability of these referral systems. As our study focused specifically on trust in primary healthcare services, and as health centres’ community-level reputation reflected on institutional aspects of trust to a limited extent, a separate assessment of systems trust in broader health services may, therefore, be better suited as an alone-standing and complementary concept rather than an integrative component of trust in primary healthcare.

In summary, the main qualitative themes reflecting on the components of trust did not only underline that the concept requires local grounding to enable meaningful conversations and analysis and also that different socioeconomic strata were likely to experience trust fundamentally differently. The following quantitative component in our mixed-method study describes the systematic patterns of trust in and across the rural communities.

### Survey results

#### Instrument construction, testing and validation

**Figure 4:**
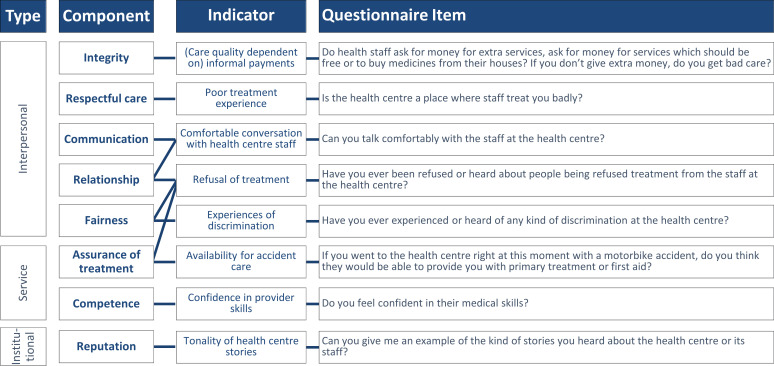
Translation of trust dimensions into measurable indicators in survey questionnaire. Source: Authors, derived from qualitative research fieldwork. Variously positively and negatively worded questions to avoid affirmation bias. Binary response options (yes/no plus ‘don’t know’) coded such that positive indications of trust (eg, a ‘no’ response to a negatively worded question) would positively contribute to the trust 8-item trust scale.

Following the questionnaire development, testing and piloting process, we arrived at an agglomerative trust index that translated the eight components of trust into concrete indicators and questionnaire items—summarised in figure 4. The interconnectedness of these components in people’s lived experiences meant that some indicators could speak to more than one dimension. For instance, refusal of treatment would reflect on treatment assurance, but respondents would also mention that the experience of refusal reflected on their sense of fairness and that the reasons for refusal hinted at the personal relationship between villagers and health centre staff.

The cognitive interviews demonstrated that the indicators reflected relevant dimensions of trust, and that respondents were able to answer the survey questions confidently. Villagers would commonly comment that they were *happy to express and share my experiences* (woman, 21, lowland Lao, Khammouane Province, cognitive interview) and that the survey questions were *easy to answer* (man, 34, lowland Lao, Champassak Province, cognitive interview) given that *those are the problems that we face every day* (man, 30, Lamed, Bokeo Province, cognitive interview). Perhaps yet more importantly, the respondents also repeatedly stressed the more fundamental value of gathering information about trust from them. For example, a female villager in Champassak Province commented that our survey team *were the first group of people I shared* [feedback about the health centre] *with* and that she *hope*[d that] *it will create some impact* (woman, 26, lowland Lao, Champassak Province, cognitive interview). Another villager would explain that, *We do want to share our opinion* [about the health centre] *but it’s quite hard. And luckily we have your project come to our village. Therefore, I am very proud and grateful to share*,’ hoping in light of their remote location that the survey would help the health centre develop (Male, 30, Lamed, Bokeo Province, cognitive interview; note that survey insights were shared accordingly with authorities on the village, district, provincial, and central levels). (While this also suggests that reticence among the respondents was limited, among the mechanisms to elicit an open response from the participants were the use of open-ended questions to avoid social desirability biases (eg, ‘Can you give me an example of the kind of stories you heard about the health centre or its staff?’) and alterations of positively and negatively worded binary response questions (eg, ‘Can you talk comfortably with the staff at the health centre?’ vs ‘Is the health centre a place where staff treat you badly?’) to limit affirmation bias among potentially reticent respondents.)

#### Statistical findings

In this final Results section, we describe the patterns and distribution of trust across and within the 14 Lao case study communities. Table 2 begins with showing the trust index, whereby each component was normalised into a value range from (−1 to +1). Adding all eight components, the overarching index ranged accordingly from −8 to +8).

**Table 2:** Distribution of trust index dimensions across Lao provinces

Component		Indicator	Province				Average
			Champassak	Bokeo	Khammouane	Xaisomboun	
Trust dimensions(−1 to +1)	Respectful care	Poor treatment	0.407	0.588	0.659	0.553	0.523
	Communication/relationship	Comfortable conversation	0.581	0.711	0.714	0.769	0.682
	Fairness	Discrimination	0.522	0.704	0.800	0.842	0.691
	Integrity (a)	Informal payments	0.554	0.485	0.323	0.783	0.573
	Integrity (b)	Payment-dependent care*	n/a	0.328	0.377	0.492	0.409
	Relationship/fairness/assurance	Refusal of treatment	0.646	0.772	0.777	0.907	0.766
	Reputation	Tonality of health centre stories	0.063	0.057	0.177	0.079	0.080
	Assurance of treatment	Availability for accident care	0.440	0.811	0.650	0.783	0.653
	Competence	Confidence in provider skills†	0.389	0.390	0.186	0.425	0.375
Overarching trust assessments		Trust index (−8 to+8)‡	4.191	4.360	4.341	4.850	4.436
		Individual assessment (−1 to +1)§	0.209	0.412	0.155	0.472	0.326
		Recommendation index (0 to +4)¶	1.029	3.252	2.703	2.882	2.286

Source: Authors.

n=1797 individuals who were aware of their local health centres (unless indicated otherwise).

*Indicator was added after pilot survey in Champassak. n=1165.

†Answer category of ‘no strong opinion’ added as neutral response option after pilot survey in Champassak.

‡Composite index of 8 trust components, using indicators of Integrity (a) in Champassak and Integrity (b) in all other provinces.

§Concluding survey question after each individual dimension, asking, ‘Overall, would you say you rather trust or distrust the healthcare centre for your personal healthcare?’ Values of [−1], [0], and [+1] correspond to responses ‘rather distrust,’ ‘neutral/no opinion/don’t know,’ and ‘rather trust,’ respectively.

¶Based on the number of times that health centres were mentioned as recommended resorts to care for (a) antenatal care, (b) place of delivery, (c) injury treatment and (d) COVID-19 care; including all respondents (n=1838) irrespective of their awareness of local health centres.

The top part of table 2 demonstrates that most indicators were on average positive (except tonality in Champassak), meaning that respondents would typically express trust rather than distrust. We can also observe a high degree of variability across the components (see ‘average’ column): The tonality of health centre stories (representing institutional trust through the community-level reputation of primary healthcare services) was the least trusting expression, followed by the perceived competence in provider skills as a form of service-related trust. In contrast, experiences of treatment refusal were relatively uncommon. Other trust dimensions with particularly low expressions were the experience of poor treatment in Champassak and Xaisomboun Provinces, common informal payments and gifts to healthcare providers in Bokeo Province, or the perceived skill level of health centre staff in Khammouane Province. The largest differences across the provinces included the presumed readiness of health centres to cater to accidents (Assurance of care), experiences of discrimination (Integrity), and treatment refusal (Relationship/Fairness/Assurance of care). Experiences of ethnic discrimination as an expression of interpersonal trust were surprisingly uncommon compared with the qualitative data: while 7.9% (142/1797) of the sample reported instances of discrimination, only 8.1% (7/87) of the 87 explicit explanations about these situations related to ethnicity, whereas with 37.1% (30/87) most cases related to economic discrimination (based on wealth and poverty).

The eight components formed a composite trust index whose values ranged from 4.19 in Champassak Province to 4.85 in Xaisomboun Province. This composite index enabled a more nuanced and insightful understanding of trust than an overarching one-dimensional indicator (ie, whether people ‘rather trust’ or ‘rather distrust’ the health centre; see bottom part of table 2), which only had moderately positive correlation coefficient of +0.44 with the trust index. Table 2 also provides an alternative measure of ‘trust,’ based on the number of times that health centres were mentioned as recommended resorts to care for (a) antenatal care, (b) place of delivery, (c) injury treatment and (d) COVID-19 care. Ranging from (0 to +4), this alternative index exhibited its lowest value of 1.03 for Champassak Province and its highest value of 3.25 in Bokeo Province, while being only mildly positively correlated with the eight-item trust index (correlation coefficient: +0.29). These scores highlight the limitations of this alternative measure: In Champassak, many case study communities were located near better-equipped hospitals that made a visit to the health centre unnecessary, whereas in Bokeo Province, many of the communities were located far away from other healthcare facilities, which made it most plausible to recommend fellow villagers to seek care at the available health centre irrespective of how much one trusts them. The eight-item index is thus a more informative assessment of active dis-/trust in local health centres; an index based on recommendations for resorts to care would instead conflate trust with pragmatic considerations of access and availability.

The socioeconomic correlates of trust were largely indistinctive. Although the SD of the index ranged within each village from 1.88 to 3.12 (with an index value range as wide as 13 index points, that is, from (−5 to +8)), neither gender, education, wealth, age, ethnic group, mother tongue or religion exhibited a noteworthy bivariate or multivariate relationship with the trust index (not shown here; see online supplemental material table A2). The trust index varied systematically across the case study communities, among which average index scores ranged from 3.83 to 5.65. A multivariate linear regression model would indicate that 7 out of 14 village identifiers had a statistically significant association with the trust index (at the 5% level). This suggests that communities tended to share relatively homogenous experiences with their local health centres, and that health centre operations tended to be more decisive in shaping local expressions of trust.

In a final step, we estimated multivariate logistic regression models that assessed the relationship between health centre utilisation of patients and pregnant women, trust and other common explanatory variables such as gender, education and household wealth. Table 3 summarises the main findings of this analysis and shows that the trust index did not exhibit a statistically significant association with health centre access during acute illness episodes, and only a mildly positive association at the ten-percent-level emerged for women seeking antenatal care during a current or recent pregnancy. While the coefficient estimates were only mildly sensitive to the inclusion of village dummy variables, overall model fitness for the smaller sample models of antenatal care was affected by their inclusion (as the removal of perfect predictors would reduce the effective sample size).

**Table 3:** Regression results

Behaviour	Acute illness: access to health centre				Antenatal care: access to health centre			
Model specification	Main results	No village dummies	Recommendation index	Recommendation index, no district dummies	Main results	No village dummies	Recommendation index	Recommendation index, no district dummies
Model number	(1)	(2)	(3)	(4)	(5)	(6)	(7)	(8)
Trust index (−8 to+8)*	0.079	0.039			0.138	0.146^*^		
	(−0.04 to 0.20)	(−0.05 to 0.13)			(−0.04 to 0.32)	(−0.02 to 0.31)		
Recommendation index (0 to+4)†			0.386^***^	0.728^***^			1.364^***^	1.046^***^
			(0.11 to 0.66)	(0.51 to 0.94)			(0.66 to 2.07)	(0.54 to 1.55)
Gender (1=female)‡	0.201	−0.069	0.251	0.114				
	(−0.47 to 0.87)	(−0.58 to 0.45)	(−0.42 to 0.92)	(−0.45 to 0.68)				
Age (in years)	−0.007	−0.015	−0.007	−0.015	−0.041	−0.016	−0.058	−0.015
	(−0.03 to 0.02)	(−0.03 to 0.00)	(−0.03 to 0.02)	(−0.04 to 0.01)	(−0.13 to 0.05)	(−0.09 to 0.06)	(−0.15 to 0.04)	(−0.09 to 0.06)
Education (number of completed years)	0.020	−0.036	0.019	−0.037	−0.008	−0.045	−0.024	−0.057
	(−0.08 to 0.12)	(−0.11 to 0.04)	(−0.08 to 0.11)	(−0.12 to 0.04)	(−0.14 to 0.13)	(−0.16 to 0.07)	(−0.17 to 0.12)	(−0.18 to 0.07)
Majority lowland Lao ethnicity (1=yes)§	−0.539	−2.253^***^	−0.379	−1.494^***^	−0.779	−1.694^***^	−1.226	−0.172
	(−1.51 to 0.43)	(−2.81 to –1.69)	(−1.38 to 0.62)	(−2.09 to –0.90)	(−3.02 to 1.46)	(−2.85 to –0.54)	(−3.73 to 1.28)	(−1.30 to 0.95)
Wealth index (0 to +1)¶	0.520	−1.073	0.297	−0.63	1.783	2.665^**^	2.611	3.867^***^
	(−1.25 to 2.29)	(−2.35 to 0.21)	(−1.47 to 2.07)	(−2.02 to 0.76)	(−1.33 to 4.90)	(0.04 to 5.28)	(−0.93 to 6.15)	(0.99 to 6.74)
Chronic disease (1=yes)^**^	0.424	0.584^**^	0.327	0.472	0.626	0.426	0.526	0.169
	(−0.25 to 1.10)	(0.04 to 1.13)	(−0.34 to 0.99)	(−0.11 to 1.05)	(−1.19 to 2.44)	(−1.22 to 2.07)	(−1.49 to 2.54)	(−1.57 to 1.91)
Self-assessed illness severity: moderate (baseline: mild)	0.021	0.069	−0.019	−0.084				
	(−0.78 to 0.82)	(−0.57 to 0.71)	(−0.82 to 0.79)	(−0.77 to 0.61)				
Self-assessed illness severity: severe (baseline: mild)	0.388	0.225	0.437	0.420				
	(−0.60 to 1.38)	(−0.56 to 1.01)	(−0.57 to 1.44)	(−0.44 to 1.28)				
Illness of adult/child (1=child supervised by respondent)	0.910^***^	0.628^**^	0.923^***^	0.607^**^				
	(0.29 to 1.53)	(0.14 to 1.12)	(0.30 to 1.54)	(0.09 to 1.13)				
Constant	−2.956^**^	0.386	−3.218^***^	−1.592^*^	1.424	0.916	1.243	−1.489
	(−5.36 to –0.55)	(−1.08 to 1.85)	(−5.64 to –0.80)	(−3.28 to 0.09)	(−2.46 to 5.31)	(−1.39 to 3.23)	(−2.62 to 5.11)	(−3.99 to 1.01)
N	409	409	421	421	105	139	108	143
Log likelihood	−152.624	−210.009	−153.307	−188.318	−53.345	−61.140	−48.128	−56.688
Chi^2^	243.952	129.183	254.464	184.443	10.847	11.693	27.356	28.059
Model test (p-value)	<0.001	<0.001	<0.001	<0.001	0.698	0.069	0.017	<0.001

Source: Authors.

Analysis on individual level, given that respondents could report one acute illness episode and/or one current/recent pregnancy each. Coefficients reported; 95% CIs in brackets. Village dummy coefficients for models 1, 3, 5 and 7 omitted for brevity. Number of observations varied because perfect predictors have been removed from analysis. Other behaviours (not reported here) would include access to other formal and informal healthcare providers as well as episodes without any healthcare access.

*p < 0.1, **p<0.05, ***p<0.01.

*Composite index of 8 trust dimensions, using indicators of Integrity (a) in Champassak and Integrity (b) in all other provinces.

†Based on the number of times that health centres were mentioned as recommended resorts to care for (a) antenatal care, (b) place of delivery, (c) injury treatment and (d) COVID-19 care; including all respondents in the full sample (n=1838) irrespective of their awareness of local health centres.

‡No respondent disclosed a non-binary gender identity. Variable omitted in antenatal care models as only women sought care (currently pregnant and recently pregnant women who delivered a child within the last 12 months prior to the survey interview).

§Lowland Lao ethnicity represents relative majority ethnic group with 38.0% of respondents in full sample (n=1830 disclosed responses). Ethnic minority groups include, among many others, Hmong (19.5%), Khmu (13.4%) and Lamed (11.9%).

¶Simple aggregate index of 8 common household assets (seven types of items including cars and rice cooker plus ratio of [>1] for number of rooms per household member).

**Data based on self-reported chronic conditions.

**Figure 5:**
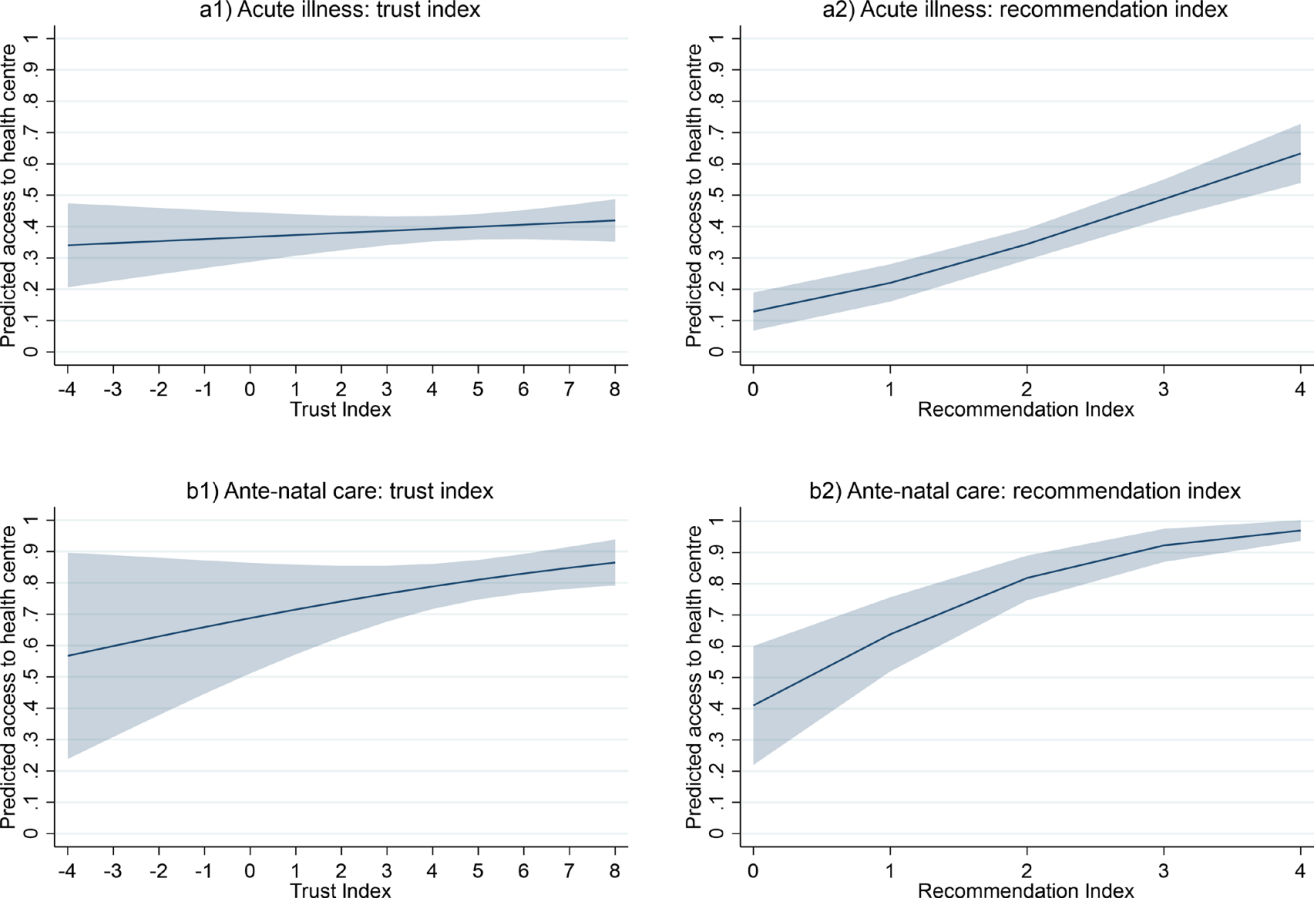
Predicted relationship between trust and health centre access Source: Authors. Predictions within index value ranges of respective sub-samples. Panels a1, a2, b1 and b2 based on Models 2, 4, 6 8 in table 2, respectively. The estimated relationship between trust index and health centre access in Panel a1 is not statistically significant.

Robustness checks using the alternative, recommendation-based index (models 3, 4, 7 and 8) exhibited a consistently positive and statistically significant association (at the 1% level). In light of the aforementioned discussion, the analysis did not discern a statistically significant relationship between trust and health centre access during acute illness episodes, whereas availability of facilities appeared to be a more decisive predictor. However, in the case of pregnancy care, trust was weakly associated with health centre utilisation (as far as the small subsamples and the cross-sectional study design allows us to infer). For example, a 1 SD increase from the mean value of the recommendation index would be associated with a 20.6% percentage-point higher predicted rate of health centre utilisation; 1 SD changes from the mean index values for recently/currently pregnant women would be associated with a 4.9% higher predicted health centre utilisation rate for the trust index and a 10.9% higher predicted rate for the recommendation index. Figure 5 summarises these relationships graphically.

## Discussion

Our qualitative research established that issues of trust were pervasive in villagers’ narratives surrounding healthcare experiences on the primary care level. Villagers were ready to articulate what trust means, and our qualitative analysis identified eight themes representing important components of trust: communication, respectful care, relationship, fairness, integrity (all as expressions of interpersonal trust), reputation (institutional trust), assurance of treatment and competence (service-related trust). We translated these components into standardised survey items to trace the expressions of trust within and across 14 Lao communities in four provinces. Cognitive interviews attested that the survey instrument resonated with local notions of trust that were important from the perspective of the rural populations. The trust index thus enabled us to compare the expressions of trust in different regions of Lao PDR, whereby individual differences appeared less pronounced than variations across health centres that cater to the communities (similar to findings in Helfinstein *et al.* 36). Analysing the relationship between trust and health centre utilisation further suggested that different levels of trust were weakly linked to pregnant women’s antenatal care choices, and robustness checks indicated that availability of healthcare providers (gauged through villagers’ recommendations for care outlets) may be a more decisive predictor of health centre access.

In comparison to studies from high-income contexts (eg, 12), our research underlined the persistent importance of interpersonal, institutional (esp. health provider reputation) and service-related types of trust between community members and primary healthcare providers,^8 9^ whereby individual dimensions of ‘confidentiality’ or ‘systems trust’ did not materialise as salient themes. Our work also provides methodological guidance on the translation from the qualitative identification of trust dimensions to their standardised assessment in a grounded and locally relevant survey instrument. In relation to the Lao research landscape in particular, our study did not detect a strong association between trust and healthcare utilisation^19 20^ but it echoed widespread concerns about informal payments^21^ and the importance of respectful care in the context of maternal and child healthcare services.^22–24^

However, further research will benefit from exploring expressions of trust across low and middle-income countries more broadly to arrive at an authoritative and grounded framework of trust to guide global health policy. Future research would also benefit from studies of trust in other informal as well as formal elements of the health system (such as traditional healers) and how health and development interventions alter this landscape with potentially unintended consequences.^37–39^

In the case of Lao PDR, our index suggested that priority areas to improve community trust in primary care services were service-related areas of provider skills and their tools, and interpersonal aspects of trust relating to practices of respectful care and the pervasive requirement of informal payments. The reputation of health centres was a key institutional element as well, whereby activities to boost the image of local health centres could include quality assurance initiatives for health centre staff communication (eg, through supportive supervision approaches) and relationship-building community outreach activities. The data analysis further suggested that the availability of healthcare providers may be more decisive in acute care situation as well as for antenatal care uptake. However, trust appears to be a factor for antenatal care access (where elements of individual choice may be more strongly emphasised than in acute care situations, see, eg, Phommachanh *et al* 22), which policy action could address potentially more economically as a first step.

### Conclusion

Trust matters in global health, but the development of the concept has lagged behind its rhetorical use in policy and research. Our study aimed to contribute to this development from a rare lower middle-income country perspective. As we demonstrated that trust in rural Lao PDR was intrinsically valued and relatively important for antenatal healthcare access among pregnant women, we add to the conceptual, methodological and empirical knowledge of a topic with ever-growing importance in global health policy and research. The practical consequence of our work is that supply-sided aspects of frontline health system development remain important but relational dimensions in health system development expressed in interpersonal, institutional and service-related trust also require explicit recognition and integration into health policies and initiatives,^40 41^ which can practically be supported by elevating the status of community engagement as a positive (ie, non-blaming) tool to foster trust and forge relationships between the general population and health service providers.^26 40 42^ In addition, trust-building interventions may not yield impact if they fail to address locally important dimensions that require improvement. As a result, top-down approaches to community engagement are likely to fail and require instead substantial groundwork with target populations to identify key dimensions and issues in their trust towards health services.^26 43^

## Data Availability

Data are available upon reasonable request. The datasets generated and analysed during the current study are not publicly available to protect the anonymity of our respondents but are available from the corresponding author on reasonable request.
